# Unfolding Thermodynamics of Cysteine-Rich Proteins and Molecular Thermal-Adaptation of Marine Ciliates

**DOI:** 10.3390/biom3040967

**Published:** 2013-11-18

**Authors:** Giorgia Cazzolli, Tatjana Škrbić, Graziano Guella, Pietro Faccioli

**Affiliations:** 1 Physics Department, Trento University, Via Sommarive 14, Povo (Trento) 38123, Italy; E-Mails: cazzolli@science.unitn.it (G.C.); guella@science.unitn.it (G.G.); faccioli@science.unitn.it (P.F.); 2 Trento Institute for Fundamental Physics and Applications (TIFPA), Via Sommarive 14, Povo (Trento) 38123, Italy; 3 International School for Advanced Studies (SISSA/ISAS), Via Bonomea 265, Trieste 34136, Italy; 4 Physics and Astronomy Department, Padua University, Via Marzolo 8, Padua 35131, Italy; 5 CNR, Institute of Biophysics, Via alla Cascata 56/C, Povo (Trento) 38123, Italy; 6 European Centre for Theoretical Nuclear Physics and Related Areas, Strada delle Tabarelle 286, Villazzano (Trento) 38100, Italy

**Keywords:** protein folding, temperature molecular adaptation, energy landscape theory, circular dichroism

## Abstract

*Euplotes nobilii* and *Euplotes raikovi* are phylogenetically closely allied species of marine ciliates, living in polar and temperate waters, respectively. Their evolutional relation and the sharply different temperatures of their natural environments make them ideal organisms to investigate thermal-adaptation. We perform a comparative study of the thermal unfolding of disulfide-rich protein pheromones produced by these ciliates. Recent circular dichroism (CD) measurements have shown that the two psychrophilic (*E. nobilii*) and mesophilic (*E. raikovi*) protein families are characterized by very different melting temperatures, despite their close structural homology. The enhanced thermal stability of the *E. raikovi* pheromones is realized notwithstanding the fact that these proteins form, as a rule, a smaller number of disulfide bonds. We perform Monte Carlo (MC) simulations in a structure-based coarse-grained (CG) model to show that the higher stability of the *E. raikovi* pheromones is due to the lower locality of the disulfide bonds, which yields a lower entropy increase in the unfolding process. Our study suggests that the higher stability of the mesophilic *E. raikovi* phermones is not mainly due to the presence of a strongly hydrophobic core, as it was proposed in the literature. In addition, we argue that the molecular adaptation of these ciliates may have occurred from cold to warm, and not from warm to cold. To provide a testable prediction, we identify a point-mutation of an *E. nobilii* pheromone that should lead to an unfolding temperature typical of that of *E. raikovi* pheromones.

## 1. Introduction

**Figure 1:**
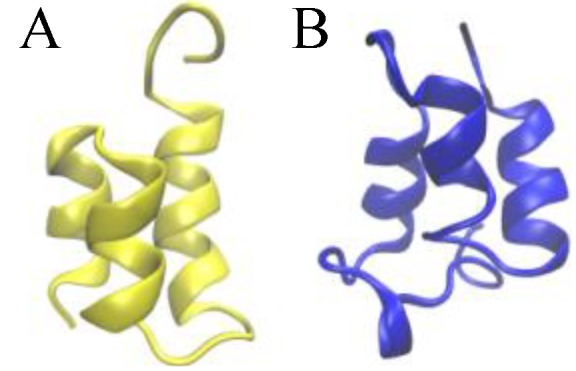
Ribbon presentations of the three-dimensional native structure of the E*r*-1 (**A**) and E*n*-1 (**B**) pheromones.

To survive in a permanently cold environment, psychrophilic microorganisms synthesize proteins that are resistant to cold-induced denaturation and misfolding [1]. On the other hand, the native states of these proteins are often only marginally stable, or even unstable in temperate environments. This case clearly illustrates how, in order to understand at the molecular level the principles that regulate the adaptation of microorganisms to different thermal environments, it is crucial to identify the general physical principles that shape the structure of the proteins’ free-energy landscapes. From this perspective, it is particularly useful to compare the folding thermodynamics of homologous proteins produced by species which are evolutionarily closely related, yet ecologically separated, such as are species living, one, in polar waters and, the other one, in temperate waters. In this way, it appears to be easier, at least in principle, to identify specific structural features that are more directly responsible for the thermodynamic stability of the protein native state. In this work, we performed a theoretical analysis of protein unfolding/refolding thermodynamics, that complements a recent analysis of circular dichroism (CD) spectra [2] of two closely homologous families of water-borne signaling proteins (known as pheromones), which regulate the vegetative (mitotic) growth and sexual mating [3] in two ecologically separated *Euplotes* species, *E. raikovi* (mesophilic) living in temperate waters and *E. nobilii* (psychrophilic) living in polar (Antarctic and Arctic) waters [4]. A representative set of members of these two families of pheromones and their corresponding Protein Data Bank (PDB) codes are listed in Table 1, while the three-dimensional structures of one *E. raikovi* pheromone and one *E. nobilii* pheromone are shown in Figure 1.

**Table 1:** List of the E*n* and E*r* pheromones investigated and corresponding PDB codes.

	*Euplotes raikovi*			*Euplotes nobilii*		
Name	Er-1	Er-2	Er-10	En-1	En-2	En-6
PDB code	1erc	1erd	1erp	2nsv	2nsw	2jms

**Figure 2:**
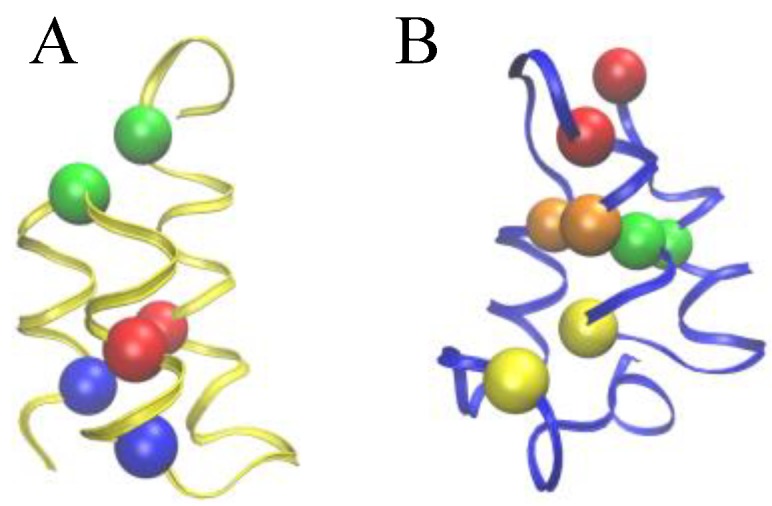
Comparison of the Cys-Cys bond patterns in the native E*r*-1 (**A**) and E*n*-1 (**B**) pheromone structures. Spheres of identical color indicate cysteine residues paired together into a disulfide bond.

The native structure of *E. nobilii* and *E. raikovi* pheromones is equally characterized by densely spaced disulfide bonds (three in *E. raikovi*, and four in *E. nobilii*), which connect residues located in different alpha-helices (see Figure 2). In a non-reducing environment at physiological temperatures, disulfide bonds provide unbreakable topological constraints [5], which strongly restrict the conformational space accessible to the polypeptide chain. The impossibility of breaking a disulfide bond at room temperature and in non-reducing conditions motivated a vast research activity directed to clarify how cysteine-rich proteins realize and possibly re-arrange the network of topological constraints leading to the correct native topology, during the folding reaction [6]. In particular, the thermodynamics for an helix-coil transition inside protein loops induced by pairs of disulfide bonds was extensively investigated, starting from the pioneering work of Scheraga and Poland, in the mid 1960s. It was realized that the presence of such covalent bonds modifies the melting curve, leading to a higher melting temperature [7,8], as expected from general polymer physics considerations [9]. In particular, it was observed that disulfide bonds between Cys residues that are sequentially distant have a larger impact on thermodynamics than those that are sequentially close.

The folding kinetics of cysteine-rich proteins was investigated in detail by Camacho and Thirumalai in the mid 1990s using both coarse-grained models [8,10], and phenomenological statistical models [11]. One striking conclusion of those studies was that, despite the fact that the disulfide bonds greatly restrict the available configuration space, it can lead to slower folding kinetics. In addition, a phenomenological scheme (the so-called proximity rule) was proposed to describe the dynamics of formation and rearrangement of disulfide bonds, during the folding reaction. From the experimental side, techniques have been developed to trap disulfide-bonded intermediates along the folding processes [12,13,14,15,16]. Another approach to experimentally assess the role of disulfide bonds is to unfold a protein while keeping them intact [17]. Disulfide cross-linking techniques have also been used in combination with mutagenic experiments in order to gain information on the folding pathways in a larger class of proteins, by substituting specific residues with cysteines [18].

In this paper, we investigate the implication of these studies on the thermodynamics of cysteine-rich proteins on the molecular adaptation of the *E. nobili* and *E. raikovi* pheromones. In a recent paper [2], it has been reported that, despite their high degree of structural similarity (homology), the *E. nobilii* pheromones (designated E*n*-1, E*n*-2 and E*n*-6) and *E. raikovi* pheromones (designated E*r*-1, E*r*-2, E*r*-10, E*r*-11, E*r*-22 and E*r*-23) are characterized by very different unfolding/refolding thermodynamics. By comparing the temperature-dependent CD spectra of these pheromones it was found that while the *E. nobilii* psychrophilic pheromones undergo an unfolding transition in temperature ranges from 55 °C to 70 °C, the mesophilic *E. raikovi* pheromones essentially remain in their native conformation up to 100 °C.

Although this difference is not surprising from an evolutionary perspective, because these pheromones are produced by organisms adapted to very different thermal environments, it is remarkable from a biophysical perspective. Indeed, a major implication of the energy landscape theory of protein folding [19] is that the folding kinetics and thermodynamics is shaped mostly by the interactions which are present in the native state and, hence, by the native tertiary structure and topology. The smoothness and funnel-like approximation of the folding landscape, which is the basis of the energy landscape theory, is supported by the successfulness of the topology-based, *i.e.*, native-centric, Gō-type or structure-based models, the terms that are used interchangeably in literature [20]. On the other hand, the primary amino acid sequence and, in general, non-native interactions are expected to play a subsidiary role (an exception, however, may be represented by proteins with knotted native topology [21,22]). In particular, a minimally-frustrated and native-centric picture of protein folding predicts that homologous proteins have closely comparable unfolding temperatures. 

The observed thermodynamics of the *E. nobilii* and *E. raikovi* pheromones challenges the native-centric view, as it appears to be dominated by effects associated to the protein primary structure and non-native interactions. In line with this new concept, it has been observed that the physicochemical properties of the polypeptide chain of cold-adapted *E. nobilii* pheromones are characterized by a reduced hydrophobicity and improved backbone flexibility [23]. Similarly, the stability of mesophilic polypeptides has been argued to be due to the presence of hydrophobic clusters [24].

In this work, we challenge this explanation by showing that the body of experimental data on the thermal unfolding of the *E. nobilii* and *E. raikovi* pheromones can be understood in terms of physical effects entirely associated to the protein native structure and topology, *i.e.*, without relying on solvent-induced effects and/or non-native interactions. In particular, we show that the unfolding temperatures of these cysteine-rich proteins are strongly influenced by the precise topology of the disulfide bonds in the protein three-dimensional native structure.

These conclusions are reached by analyzing the results of an extensive Monte Carlo (MC) simulations of the conformational space sampled by the *E. nobilii* and *E. raikovi* pheromones, performed at different temperatures, within a purely native-centric Gō-type model implemented on the coarse-grained (CG) level. In this approach, attractive non-bonded interactions are assigned only to the pairs of amino-acids that are in contact in the native state. Further, because sulfur bridges impose topological constraints that cannot be broken in the wide temperature range of interest (in the absence of chemical denaturants), we have modeled them appropriately in order to assure they remain unbreakable at room temperature. Indeed, the strength of disulfide bond is about 60 kcal/mol*,* *i.e.*, of the order of 10 *RT*, at the temperature of *T* = 300 K. We also emphasize that this model is completely blind to any physical effect associated to the primary structure of the considered polypeptide chains.

Despite its simplicity, this model reproduces well the observed differences in the unfolding temperatures between the members of the two pheromone families. In particular, in our simulations, the *E. raikovi* pheromones remain native over the entire temperature range that has been considered, while the *E. nobilii* pheromones unfold at a (nominal) temperature which ranges from 40 °C to 60 °C. These results strongly suggest that the thermodynamics of the two pheromone families is driven by the structural properties of the protein native states, rather than by physical effects associated to the specificities of the amino-acid sequences. Using an analytically solvable statistical model, we argue that the much stronger relative stability of *E. raikovi* pheromons with respect to *E. nobilii* pheromones is due to the higher non-locality of the Cys-Cys bonds. 

These conclusions are also corroborated by the behavior of the fractional helicity (mole fraction of helical backbone within the peptide or protein) of the *E. nobilii* E*n*-1 and *E. raikovi* E*r*-1 pheromones at different temperatures. We find that our theoretical predictions agree well with the corresponding experimental results for the main secondary structural motifs, which were estimated by deconvoluting the whole experimental CD spectra using the DICHROWEB web interface.

## 2. Results and Discussion

**Figure 3:**
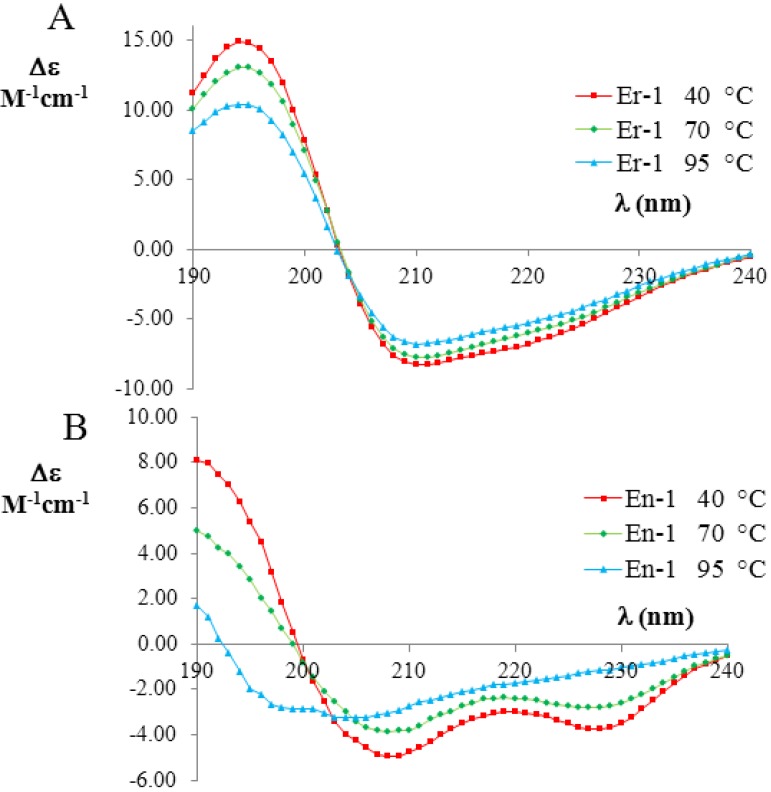
Far-UV circular dichroism (CD) spectra (expressed in *Δε* units) of the mesophilic, E*r*-1 (**A**), and psychrophilic, E*n*-1 (**B**), pheromones (20 mM, pH 6) at three selected temperatures (red 40 °C; green 70 °C; and blue 90 °C). The raw CD data were kindly supplied by M. Geralt and reported in [2].

To quantify the amount of unfolding of secondary structures in the two pheromone families, we used the raw CD data reported in Geralt *et al*. [2] to evaluate the temperature dependence of the fractional helicity *f_H_* for two representative polypeptide chains, *i.e.*, E*n*-1 and E*r*-1. The temperature dependent CD measurements of E*n*-1 and E*r*-1 pheromones (expressed as the difference in the molar extinction coefficients; for more details see Experimental Section) are shown in Figure 3A,B, respectively. The far-UV CD spectra of E*r*-1 showed a strong negative Cotton effect at 208 nm and 222 nm, thus indicating that the *α*-helix conformations provide a relevant contribution to the overall secondary structure of this protein. The deconvolution of the E*r*-1 CD spectra obtained at 40 °C and 70 °C from the DICHROWEB application, indicated a high and similar helix content *f_H_* = (70 ± 8)%, in a good agreement with E*r*-1 NMR [25] and X-ray analysis [26,27,28] in which the contribution to helical structures was estimated to be 62% and 67%, respectively. Moreover, from the deconvolution of the E*r*-1 CD spectrum at 95 °C, the fraction of residues involved in *α*-helical structures was found to remain high, *i.e.*, (53 ± 3)%. Overall, within the wide temperature range 20–100 °C, the *f_H_* parameter decreases less than 25%. 

In contrast, the deconvolution of the corresponding E*n*-1 CD spectra in the same temperature range lead to a strikingly different result. At 40 °C, the helical contribution was measured to be in rough agreement *f_H_* = (57 ± 8)% with the value coming from NMR analysis, where it was estimated to be 46% [23,29]. However, the *f_H_* values measured from the deconvolution of the CD spectra at 70 °C and 95 °C were markedly lower ((41 ± 14)% and (16 ± 7)%, respectively) than at 40 °C, implying that there is a significant loss (by a factor greater than three) in *α*-helix structures.

Let us now compare this body of experimental results with the results of the MC simulations performed in the native-centric CG model described in the Experimental Section. To this end, it is instructive to first consider the temperature dependence of the average value of the fluctuations in the fraction of native contacts, *i.e.*,

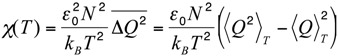
(1)
where < >*_T_* denotes the thermal average at the temperature *T*, *N* is the total number of native contacts, *Q* is the fraction of native contacts that are present in a given conformation, *ε_0_* = 2 kcal/mol is a typical contact energy and *k_B_* is the Boltzmann constant. To this regard it is worth recalling that, in the present simplified model, non-bonded attractive interactions are assigned exclusively to pairs of amino-acids that are in native contact. Hence, the definition in Equation (1) basically coincides with a rescaled specific heat, since it includes only the contributions from the statistical fluctuations of the native interactions, which are directly correlated with the unfolding transition, and hence drive the characteristic peak in the specific heat *versus* temperature curve. On the other side, the statistical fluctuations coming from repulsive hard-core interactions are quite insensitive to the unfolding transition. Indeed, due to topological constraints imposed by sulfur bridges, the chain remains in a compact state, even at high temperature. Consequently, the hard-core interactions introduce a flat background in the specific heat curve, which makes it difficult to identify the unfolding transition temperature. In order to remove this background and emphasize the differences in the thermodynamics of the two pheromone families, we have chosen to analyze the rescaled specific heat given by Equation (1). Therefore, the unfolding transition temperature can be identified by a peak in the *χ(T)* curve. We also note that since the disulfide bridges are unbreakable in the temperature range of interest, the observed peaks are not as sharp as in the case of small globular proteins of the same size with no cysteine bridges at all, e.g., protein-G [33].

**Figure 4:**
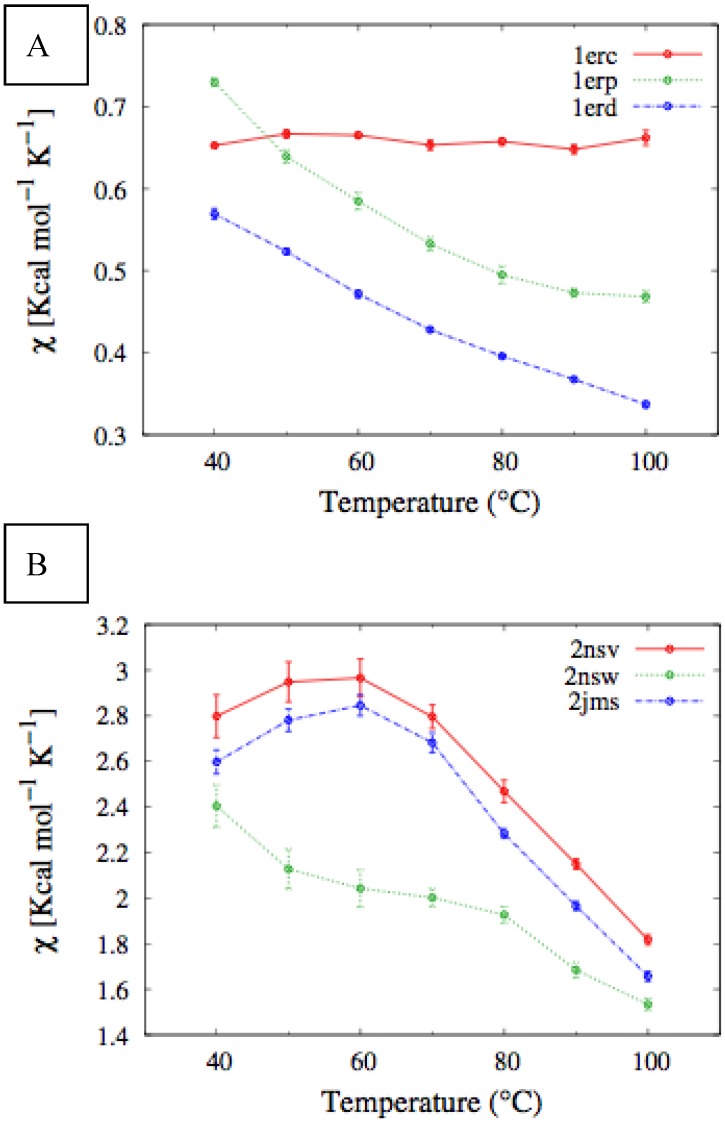
Comparison of the temperature-dependence of the fluctuation of fraction of native contacts defined in Equation (1) for different E*r* (**A**) and E*n* (**B**) pheromones obtained by means of MC simulations in the CG model defined in the Experimental Section.

**Figure 5:**
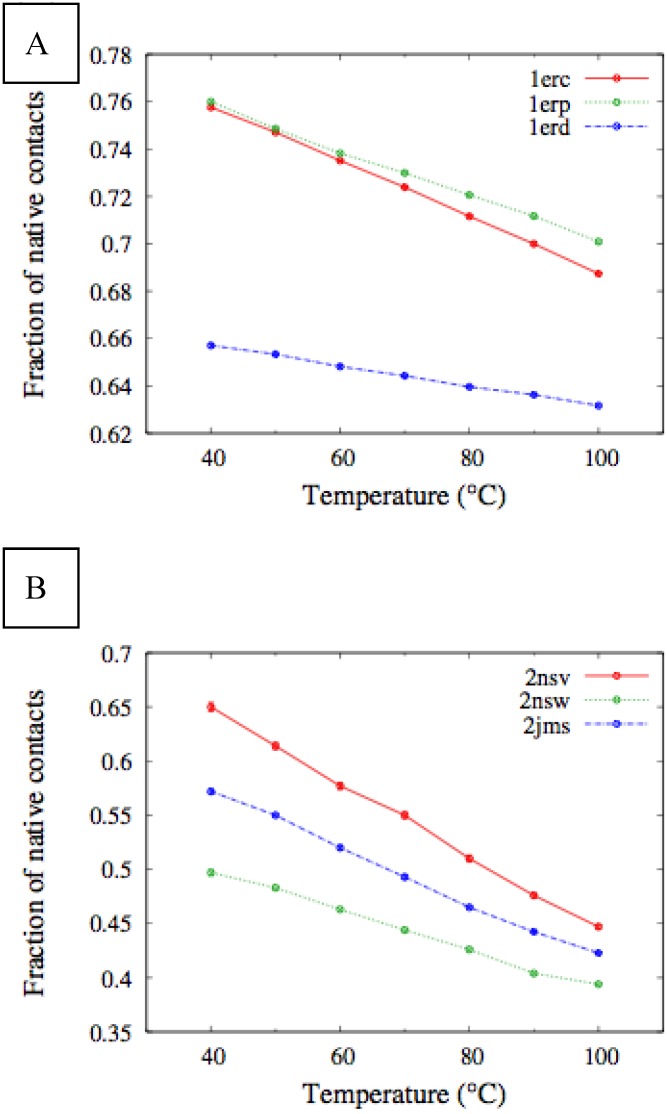
Temperature dependence of the fraction of native contacts for different E*r* (**A**) and E*n* (**B**) pheromones, obtained by means of MC simulations in the CG model defined in the Experimental Section.

In Figure 4, we report the values of *χ(T)* at different temperatures for the polypeptide chains belonging to the *E. nobilii* and *E. raikovi* pheromone families, respectively, while in Figure 5 we show the corresponding average fraction of native contacts. It appears that *E. nobilii* pheromones are characterized by an unfolding transition in the temperature range considered. Remarkably, no unfolding peak in the fluctuation of native contacts is shown by any of the simulated *E. raikovi* pheromones. In addition, we emphasize that the magnitude and the temperature dependence of the fraction of native contacts in *α*-helixes computed from our MC simulations qualitatively resembles those of the helical fraction *f_H_*, which was extracted directly from the deconvolution of the CD data. Indeed, both quantities show an overall smaller helical content, as well as steeper secondary motif suppression with temperature, in the *E. nobili* pheromones.

**Figure 6:**
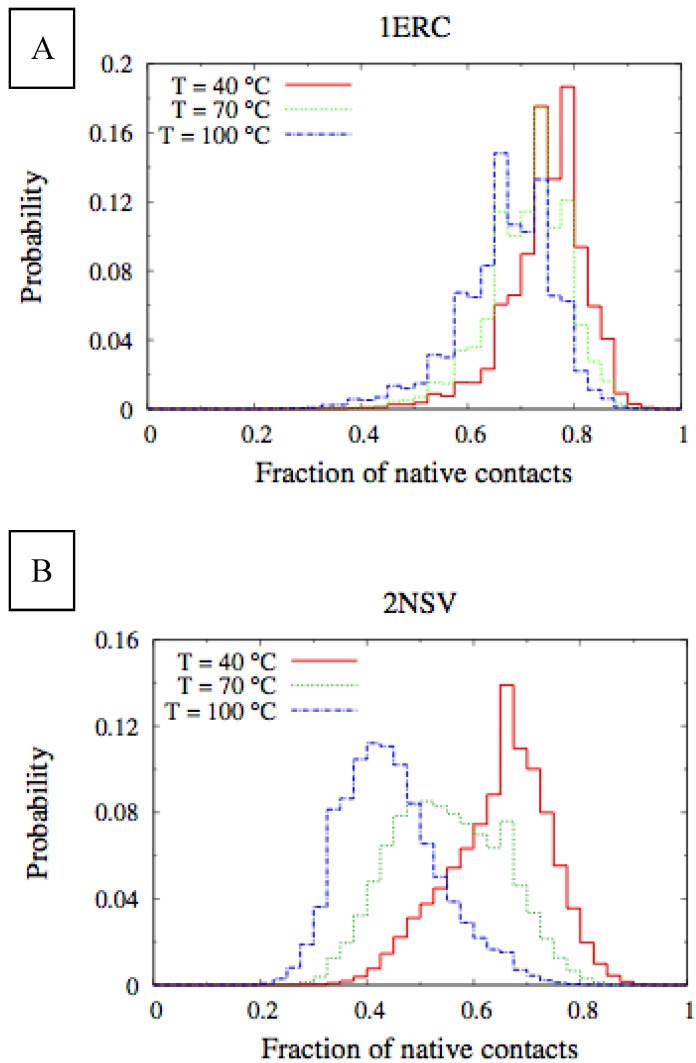
Equilibrium distributions of the fraction of native contacts at different temperatures for the E*r*-1 (**A**) and E*n*-1 (**B**) pheromones.

In Figure 6, we compare the equilibrium distributions of the fraction of native contacts in two representative pheromones, namely E*r*-1of *E. raikovi* and E*n*-1 of *E. nobilii*, at different temperatures. In E*r*-1, this distribution appears to be almost insensitive to temperature variations and the protein remains native even at the highest temperature considered. On the other hand, the fraction of native contacts in the E*n*-1 polypeptide chain drops significantly at high temperatures. These results match even at a semi-quantitative level the distributions shown in Table 2, which were obtained by deconvoluting the CD experimental data. In principle, the model’s parameters may be adjusted to in order to reproduce quantitatively also the experimentally observed relatively low helicity of the high-temperature experiments (close to 100 °C), e.g., by slightly decreasing the strength of the native interactions involved in the secondary structures. However, the main point of this analysis was to show that such a model allows for rationalization of the mechanism behind the qualitative difference in the thermodynamics of the two pheromone families. For this reason we choose to avoid the fine tuning of the parameters, thereby allowing for this small discrepancy.

**Table 2:** Values of fractional helicity *f_H_* at different temperatures for the E*r*-1 and E*n*-1 proteins, obtained by DICHROWEB/CONTINLL-4 analysis. The values in parenthesis are the corresponding normalized root mean square deviations. The last column reports the values of fractional helicity *f_H_* of E*r*-1 and E*n*-1 as established by X-ray [27] and NMR [23], respectively.

Pheromone	*f_H_* (CD, 40 °C)	*f_H_* (CD, 70 °C)	*f_H_* (CD, 95 °C)	*f_H_* reported
E*r*-1	0.72 (0.07)	0.68 (0.08)	0.53 (0.03)	0.678 (X-ray)
E*n*-1	0.57 (0.08)	0.41 (0.14)	0.16 (0.07)	0.46 (NMR)

These results overall indicate that our simple CG model, which only encodes the information related to three dimensional native structure and the location of the disulfide bonds, is able to closely reproduce the observed difference in the pheromone unfolding/refolding thermodynamics. We emphasize that no parameter in the energy function described in the Experimental Section was fit in order to produce the observed differences between the E*n* and E*r* pheromones. 

It is important to point out that the energy function of the CG model encodes only the information related to the protein tertiary structure and the location of the Cys-Cys bonds. Therefore, the remarkable agreement that we find with the experimental data suggests that the specificities of the chemical composition of the polypeptide chain and the non-native interactions do not significantly affect the thermodynamics of these systems. This finding contrasts with the explanation of the enhanced thermal stability of the E*n* pheromones based on the existence of a strongly hydrophobic cluster, which was proposed [23]. 

In general, it is difficult to justify the observed large effects on the unfolding temperatures in terms of the relatively small differences in the three-dimensional crystal structures of the different pheromones. In view of the results of the theoretical studies on the thermodynamics of cysteine-rich proteins [7,8,10,11], we argue that the observed differences are instead generated by the different topological constraints that are imposed by the specific location of the disulfide bonds.

To substantiate this hypothesis, we analyzed the localization of the pheromone hydrogen-bonded and disulfide-bonded native contacts, and compared the contact order (*CO*) of the pheromone native structures in accordance with the parameters provided by [30] where,

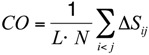
(2)


In this formula, *N* is the total number of contacts, *L* is the total number of amino acids in the protein, and *ΔS_ij_* is the sequence separation between contacting residues *i* and *j*. The *CO* values of all the *E. nobilii* and *E. raikovi* pheromones are reported in Table 3. It appears that all the polypeptide chains have *CO* values close to 0.3, which is a typical value for *α*-helix proteins. Hence, the localization of generic tertiary contacts cannot be at the origin of the observed large difference in the unfolding temperature. 

**Table 3:** The contact order calculated using all native contacts and the contact order calculated only for Cys-Cys native contacts are reported for the investigated pheromones.

	*Euplotes raikovi*			*Euplotes nobilii*		
Name	Er-1	Er-2	Er-10	En-1	En-2	En-6
*CO_total_*	0.19	0.19	0.19	0.22	0.19	0.21
*CO_Cys-Cys_*	0.46	0.43	0.48	0.34	0.32	0.32

On the other hand, a significant dissimilarity between the *E. nobilii* and *E. raikovi* pheromones emerges when we compare a *CO* variant in which the sum runs only over disulfide native contacts:

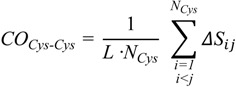
(3)


In this formula, the indexes *i* and *j* run over Cys residues only and *N_Cys_* denotes the total number of sulfur bridges. The values of this observable for all pheromones are reported in Table 3. It appears that the *E. raikovi* pheromones have *CO_Cys-Cys_* values close to 0.45, while the *E. nobilii* pheromones have *CO_Cys-Cys_* close to 0.33. This difference implies that the disulfide bonds in the mesophilic *E. raikovi* pheromones are systematically less local than in the psychrophilic *E. nobilii* pheromones. In order to illustrate how a lower locality of the disulfide bonds can imply a higher unfolding temperature, in the appendix we discuss a minimalistic statistical toy model, which can be solved analytically. 

To allow for a further validation of our theoretical explanation of the CD data, we lastly addressed the question of the minimal set of mutations capable of increasing the unfolding temperature of *E. nobilii* pheromones up to the values typical of *E. raikovi* pheromones. The basic rationale was to reposition the Cys-Cys bonds in the native structure of a representative *E. nobilii* pheromone in a way to attain the values of the contact order of cysteine-bonds that are typical of the other, that of *E. raikovi* pheromone family. In particular, we designed a mutant of the *E. nobilii* E*n*-1 protein in which two of the Cys-Cys bonds have been translated from positions 11–37 to 15–37 and from 30–52 to 27–52, and Cys residues in the positions 23 and 33 have been replaced by Gly residues. In this way, the disulfide bonds reproduce the same disulfide bond patterns of the *E. raikovi* E*r*-1 polypeptide chain (see the right panel of Figure 2). With these modifications, the new mutant protein, despite it contains one disulfide bond less, has an increased value of the contact order of sulfur bridges, and thus is expected to obey a higher thermal stability, that typical of *E. raikovi* pheromones. The primary sequences of the wild-type and mutant E*n*-1 chains are the following:

E*n*-1-wildtype:
  NPEDWFTPDTCAYGDSNTAWTTCTTPGQTCYTCCSSCFDVVGEQACQMSAQC
E*n*-1-mutant:
  NPEDWFTPDTGAYGCSNTAWTTGTTPCQTGYTGCSSCFDVVGEQACQMSAQC

**Figure 7:**
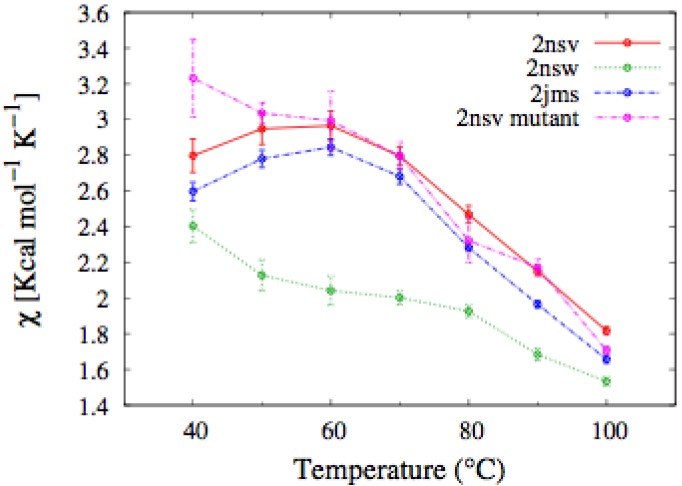
Temperature dependence of the fluctuation in the number of native contacts defined in Equation (1), for the E*n* pheromones and the mutant of the E*n*-1 chain. In the mutant, the characteristic peak signaling the unfolding transition disappears.

By assuming that such a small mutation does not affect at all the three dimensional structure of the E*n*-1 polypeptide chain, we simulated the thermodynamics of the new mutant protein in the native-centric CG model. The results are presented in Figure 7. As expected, the E*n*-1 mutant protein showed a qualitatively similar thermodynamics in respect of that of the *E. raikovii* pheromones. In particular, the temperature dependence of the average fluctuation of the fraction of native contacts, defined in Equation 1, for the mutant does not display a peak signaling the unfolding transition, which is present in the curve relative to the wilde type protein. It would be very interesting in the future to assess this simulation by direct CD measurements of this mutant protein. In concluding this section, we deserve a comment to the hypothesis, proposed by Geralt *et al.* [2], that the differences in the thermodynamic properties between the *E. nobilii* and *E. raikovi* pheromones may be due to a substantially unstructured N-terminal extension distinctive of *E. nobilii* pheromones. This hypothesis does not appear to be supported by the above observations on the enhanced stability of the E*n*-1 mutant protein, that includes an extension of 13 amino-acids at the *N*-terminal with respect to the E*r*-1 pheromone. In addition, a simulation of a second mutant protein, in which the 13 residues at the *N*-terminal were removed and the remaining residues are the same as in the first mutant, showed the thermodynamics of the E*r*-type (data not showed). 

## 3. Experimental

### 3.1. Coarse-Grained Model

Among many flavors of Gō-type models, some of which are implemented for all-atom representations [31], and the other on the different levels of coarse-graining [32], we have adopted the CG model developed by Karanicolas and Brooks [33]. In this model, the effective degrees of freedom are the amino-acid residues, represented by spherical beads located at the position of the corresponding C_α_ atom.

The energy function of this model consists of a set of bonded and non-bonded contributions. The bonded part of the potential accounts for the stretching of the pseudo-bond C_α_-C_α_ and bending of the pseudo-angle C_α_-C_α_-C_α_, as well as of the pseudo-torsions C_α_-C_α_-C_α_-C_α_:
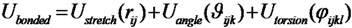
(4)

- the bond stretching has a simple harmonic spring form:

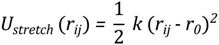
(5)

where *r_ij_* is the distance between consecutive C_α_ atoms and *r_0_*= 0.38 nm is the equilibrium distance.

- the pseudo-angle potential is given by:

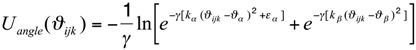
(6)

where *ϑ_ij_* is the pseudo-angle formed by the residues *i,j* and *k*, while *ϑ*_α_ = 92° and *ϑ_β_* = 130° are the equilibrium values of the helical and the extended pseudo-angles, respectively.

- the form of the torsion-angle potential is:

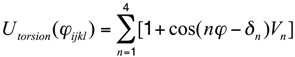
(7)

where *φ_ijkl_* is the dihedral angle between the planes identified by the position of the beeds *i*, *j*, *k* and *j*, *k*, *l* and the constants *δ_n_* and *V_n_* only on the type of residues identified by the label *j* and *k*. The non-bonded sector of the energy function accounts for the short-distance steric repulsion between all amino acids. In addition, an attractive component to the amino acid interactions is assigned to pairs of residues that are in contact in the native state. Within such an approach, the native contact map is defined based on the network of the hydrogen bonds in the native state, as well as on the degree of the proximity of the backbone atom side-chains. Namely, two residues are defined to be in the native contact if the hydrogen bond between them is stronger than −0.5 kcal/mol, or if any of their non-hydrogen side-chain atoms are within the distance of 0.45 nm in the native state. We do not consider contacts between residues with a distance in sequence smaller than 3 amino-acids. These interactions are described by the following functional form:

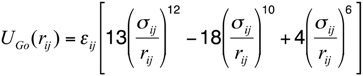
(8)
where σ_ij_ is the native separation between residues *i* and *j*. For residues that are hydrogen bonded in the native states, the strength of the interaction *ε_ij_* was chosen in such a way to match that of the corresponding hydrogen bond. For side-chain interacting residues, it is a value proportional to the corresponding Miyazawa-Jernigan contact potential [34] and it was suitably renormalized in order to match the hydrogen bond native contact energy scale. The interaction energy between Cys-Cys pairs that form native disulfide bridges was modeled employing the value 15 times larger than the corresponding Miyazawa-Jernigan contact potential between any pair of cysteine residues. This factor was derived to account for the strength of disulfide bond, so to set this value close to 60 kcal/mol. The exact value of this factor is irrelevant, as far as it keeps the native disulfide bridges unbreakable. 

All the values of the parameters appearing in the expressions of the bonded and non-bonded energy terms can be found elsewhere [33]. This model has recently been shown to generate results that compare positively with atomistic protein folding simulations for a small globular protein domain [35].

### 3.2. Monte Carlo Simulations

To sample the ensemble of chain conformations at thermal equilibrium in this model, we have used a Monte Carlo (MC) algorithm that combines different types of local and global trial moves, namely:

- crankshaft moves [36], that consist of the rotation of a randomly selected single bead around the axis defined by its nearest neighbors. The angle of the rotation was randomly selected in the interval *Δφ_max_* = ±30°;
- end-point moves, in which the last 10 residues on both terminals are rotated rigidly with respect to the rest of the chain by up to 30° around a random axis passing through the most interior bead of the end segment;
- Cartesian moves, that involve the displacement of the coordinates of a single randomly selected bead in the chain, within a sphere with radius of 0.015 nm;
- pivot moves [37], where one amino acid is picked at random and the chain portion involving all amino acids with smaller (or alternatively larger) sequence index are rotated by up to 30° around a random axis passing through the picked amino acid.

The trial moves were accepted or rejected according to the standard Metropolis criterion. The boldness and the relative probability of the different moves was set in order to have a global acceptance ratio close to 50%.

For each chain in the two families, we generated 12 independent MC trajectories, each consisting of 20,000 uncorrelated configurations. Standard autocorrelation analysis was employed. 

### 3.3. Analysis of the CD Data

Due to differential absorption produced by left- and right-handed polarized light in the far UV wavelength range (below 240 nm) in response to electronic transitions of polypeptide backbone peptide bonds in different conformations, circular dichroism (CD) is a spectroscopic technique used to determine the secondary structural content of proteins. In particular, the uniform backbone conformation and the dominant effect of the amide chromophore in the far-UV region produce characteristic CD spectra depending on the relative populations of the main secondary structural motifs, such as α-helices and extended *β*-sheets. In particular α-helix conformations are promptly detected trough the electrically forbidden (but magnetically allowed) transition *n*→ π* leading to the negative band at 220–225 nm, as well as the allowed transition π → π* which shows a strong positive component at about 190 nm and a strong negative band at about 208 nm. 

To date, the simplest methods for evaluating the fractional helicity (mole fraction of helical backbone within the peptide or protein) rely on parametric equations relating to the molar circular dichroism *Δε* value at 222 nm. Other methods use the whole experimental CD spectrum of a protein to predict its conformations and, consistently, relate to linear, or non-linear statistical methods implying best-fitting procedures with linear combinations of far-CD spectra based on reference proteins with known tertiary structure. Among these methods, we choose DICHROWEB [38,39,40]. This is a web interface that in addition to enabling calculations using various combinations of several different algorithms and reference databases, leads to reliable goodness-of-fit parameters and graphical comparisons. 

Raw CD data (mdeg/*λ* and mdeg/*T*) for the investigated E*r*-1 and E*n*-1 pheromones were kindly supplied by M. Geralt. The experimental conditions of these measurements are shortly described elsewhere [2] where also the temperature dependence of CD spectra for the pheromones E*n*-1, E*n*-2, E*n*-6, E*r*-22 and E*r*-23 have been reported. Lyophilized samples of each pheromone were dissolved in 20 mM sodium phosphate buffer, at pH 6.0, and diluted to a protein concentration of 20 *μ*M. CD experiments were recorded using the temperature/wavelength scan software supplied with the Jasco 815 CD spectrophotometer between 260 nm and 190 nm in a thermostatically controlled (20 °C) quartz cell of 0.1 cm path length. For any measurement accumulation, an average of 10 spectra were obtained with a scanning speed of 20 nm/min, a response time of 4 s, and band-width of 1 nm. For temperature dependent measurements, the temperature was raised from 5 °C to 95 °C for E*r*-1, and from 20 °C to 95 °C for E*n*-1 with 0.5 °C/min. Spectra were recorded every 5 °C (50 nm/min, 5 accumulations) 5 min after that each value of temperature was reached. 

The temperature dependent CD measurements of E*r*-1 and E*n*-1 (expressed as the difference in the molar extinction coefficients) in the far-UV region are shown in Figure 3. The best results on the de-convoluted CD spectra of the E*r*-1 and E*n*-1 pheromones (in terms of normalized root mean square deviation) were obtained by applying the algorithm CONTINLL [41,42] and the protein reference dataset 4. 

## 4. Conclusions

In this work, we presented an interpretation of the recent experimental CD results on the unfolding/refolding thermodynamics of protein pheromones isolated from the psychrophilic species, *E. nobilii*, and the mesophilic species, *E. raikovi*. CD measurements followed by DICHROWEB assisted deconvolution indicated that in the *E. nobilii* pheromones the *α*-helical content decreases drastically in the temperature range from 55 °C to 70 °C. By contrast, in *E. raikovi* pheromones this fraction remains almost unaltered even at 100 °C. Based on the results of numerical MC simulations in a CG native-centric model we are led to the conclusion that the observed enhanced stability of the *E. raikovi* pheromones is due to the non-local pattern of topological constrains determined by the disulfide bonds, which reduce the conformational entropy gain in unfolding the secondary structures. To allow for further experimental assessment of our conclusions we designed an *E. nobilii* pheromone mutant, which is expected to unfold at a very high temperature like *E. raikovi* pheromones.

On the one hand, the results presented in this work challenge the explanation originally proposed [23], according to which the enhanced thermal resistance of the *E. raikovi* is due to the presence of a strong hydrophobic core, hence to a sequence-dependent effect. On the other hand, they implicitly suggest that the thermal adaptation of these molecules occurred from the cold to the temperate waters rather than from temperate to cold waters. To our knowledge, this possibility has not been considered so far in the biology literature.

As a final remark, we point out that measurements of the NMR chemical shifts for these proteins would be extremely valuable. On the one hand, using the technique recently developed [43], they would allow to obtain an accurate experimental determination of the secondary structure content, even in the high temperature regime. On the other hand, NMR-guided meta-dynamics simulations may offer a computationally efficient scheme to complement the present theoretical results with more realistic simulations, based on atomistic force fields [44].

## Figures and Tables

**Figure A1:**
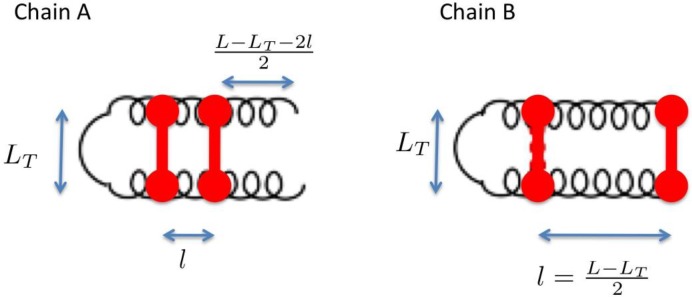
Schematic representations of the two homologous proteins considered in the analytically solvable toy-model.

## Appendix

### A Simple Statistical Toy Model Illustrating the Correlation between Delocalization of Cys-Cys Bonds and Unfolding Temperature

In order to illustrate how a higher locality of the topological constraints can induce a lower unfolding temperature it is instructive to analyze analytically-solvable statistical toy-models. The impact of unfolding entropy variations due to Cys-Cys bonds in protein loops comprising helical structure has been discussed in great detail by Scheraga and using a quite sophisticated yet analytically solvable statistical model [7]. For our illustrative purposes, however, it is sufficient to consider two much simpler toy systems, defined by the two homologous protein structures shown in Figure A1, hereby denominated *A* and *B*. Both polypeptide chains have the same length *L* and consist of *N* amino-acids organized in two symmetric helixes, linked by a turn of length *L_T_* and “pinched” by two unbreakable Cys-Cys bonds.

In both chains, one of the two disulfide bonds is located in turn region. In the protein *B*, the second disulfide bond connects the chain terminals, while in the protein *A* the second disulfide bond pinches two amino-acids located at a distance *l* from the beginning of the turn. Clearly, the topological constraints induced by the disulfide bonds are more local in chain *A* than in chain *B*. In particular the chain *A* has a lower Cys-Cys contact order.

Let us now compare the conformational entropy gains *ΔS_A_* and *ΔS_B_* for completing the unfolding the two chains, under the condition of fulfilling the topological constraints provided by the disulfide bonds. These entropy changes can be estimated analytically by neglecting the entropy of the native state and assuming that unfolded proteins can be treated as random Gaussian chains, subject to the topological constrains provided by the Cys-Cys bonds. In the limit in wich the equilibrium distance of these bonds is small compared to the persistence length of the chain we obtain the following result:

(9)

Note that the entropy difference is positive, since by definition *2l < L−L_T_*.

At the unfolding temperature, the free energy of the native state equals that of the denatured state. By denoting *T_A_* and *T_B_* the unfolding temperatures of the two chains, with *H_N_ = −|H_N_|* being the (negative) enthalpy of the native states we obtain:

(10)

that yields:

(11)

Hence, the chain *A* characterized by a higher locality of Cys-Cys bonds (hence a lower *CO_Cys-Cys_* value) has also a lower unfolding temperature. This simple analytical model, along with our numerical simulations, coherently explains the origin of the different unfolding temperatures between *E. nobilii* and *E. raikovi* pheromones in terms of locality of the disulfide bonds.
